# Comparison of Measured 24-Hour Urinary Salt Excretion With Spot Urine and 24-Hour Dietary Recall Estimates Among Adolescents and Parents: Cross-Sectional Study

**DOI:** 10.2196/85549

**Published:** 2026-06-30

**Authors:** Sandeep Kaur, Akashdeep Singh Chauhan, Rajesh Kumar, Manmeet Kaur

**Affiliations:** 1 Faculty of Medicine, Imperial College London, Hammersmith Campus, London, W120NN, United Kingdom, 44 7570297460; 2 Indian Institute of Public Health-Delhi, Public Health Foundation of India, New Delhi, India; 3 Department of Community Medicine and School of Public Health, Post Graduate Institute of Medical Education and Research, Chandigarh, India; 4 Health Equity Action Learnings Foundation, Chandigarh, India

**Keywords:** hypertension, cardiovascular diseases, urinary excreted salt levels, chronic diseases, excretory salt, 24-hour urine collection, spot urine sampling, dietary recall, adolescents

## Abstract

**Background:**

Hypertension, a leading cause of cardiovascular disease, is a significant public health challenge, with urban India reporting prevalence rates up to 40%. Excessive salt intake, averaging 8 to 11 g/day in India, far exceeds the World Health Organization–recommended limit of 5 g/day and is a key modifiable risk factor. While 24-hour urine collection is the gold standard for measuring 24-hour urinary excreted salt levels, its practicality in large-scale studies is limited, necessitating alternative methods such as spot urine sampling and dietary recall.

**Objective:**

This study aimed to address current knowledge gaps by evaluating 24-hour urinary excretory salt levels across age groups and comparing the gold standard of 24-hour urinary salt excretion with estimates from spot urine samples and 24-hour dietary recalls among adolescents and their parents in Chandigarh, India.

**Methods:**

A cross-sectional survey was conducted among 462 adolescents and 395 parents from public schools in Chandigarh, part of a cluster randomized controlled trial focused on reducing chronic disease risk factors. Twenty-four–hour urinary excreted salt levels were measured via 24-hour urine collection, spot urine samples were analyzed with the Kawasaki, Tanaka, and INTERSALT equations, and 2 nonconsecutive 24-hour dietary recalls using a correction factor of 0.9. Bland-Altman plots were used to assess agreement between methods.

**Results:**

The mean 24-hour urinary excreted salt levels measured via 24-hour urine samples were 6.95 g/day (SD 0.24; 95% CI 6.48‐7.42) for adolescents and 7.73 g/day (SD 0.3; 95% CI 7.18‐8.28) for parents. Spot urine estimates showed variability, with the INTERSALT equation with potassium yielding the closest results to 24-hour measurements for parents (mean 8.12, SD 0.4 g/d; 95% CI 7.36‐8.95) and adolescents (mean 7.97, SD 0.4 g/day; 95% CI 7.16‐8.77). Dietary recall underestimated 24-hour urinary excreted salt levels for both groups (mean 4.15, SD 0.08 g/day for adolescents and mean 3.62, SD 0.08 g/day for parents).

**Conclusions:**

Spot urine sampling, particularly the INTERSALT with potassium equation, offers a feasible alternative to 24-hour urine collection for estimating population-level 24-hour urinary excreted salt levels among adolescents and adults in resource-limited settings. Dietary recall alone significantly underestimates intake and should be supplemented with objective measures. These findings support the use of tailored estimation methods based on age for improved hypertension management strategies in India.

## Introduction

Hypertension is a major global health issue, significantly contributing to the burden of cardiovascular disease, which remains the leading cause of death worldwide [1]. In India, the prevalence of hypertension is high, with 40% of urban adults living with hypertension, compared with 17% in rural areas [2]. In Chandigarh, a union territory, the issue is particularly pronounced, affecting 45% of women and 58% of men [2].

Excessive salt intake is one of the most impactful yet modifiable risk factors for hypertension [3-5]. Globally, average salt consumption is nearly twice the maximum daily intake of 5 g/day recommended by the World Health Organization (WHO) [6]. In India, estimates indicate that people consume an average of 11 g/day, while recent national data report a mean intake of 8 g/day among adults aged 18 to 69 years [2,6]. Similar trends are seen in other countries; for instance, a study from Iran found that adolescents aged 10 to 15 years consume about 9.7 g/day, reflecting a widespread pattern of high salt intake across age groups [7]. The latest 2024 report suggests that nearly 1.4 billion people worldwide are hypertensive, underscoring the need for effective preventive and management strategies [1,8]. Studies from India and other low- and middle-income countries have shown that high salt consumption during adolescence is a risk factor for hypertension later in life [5,9].

To create effective management strategies, it is important to have clear criteria for evaluating the impact of different approaches. While the health risks linked to high salt intake are well known, accurately measuring salt consumption in the population is limited by several challenges. These difficulties make it hard to obtain reliable data on salt intake patterns, which in turn affects the development and implementation of targeted public health programs aimed at reducing health risks [10]. While the 24-hour urine collection method is the most accurate for estimating 24-hour urinary excretory salt levels, it is often impractical for large population studies due to logistical demands. Spot urine sampling provides a more feasible alternative, using formulas such as INTERSALT, Kawasaki, and Tanaka to estimate 24-hour urinary salt excretion from single urine samples or through 24-hour dietary recall [10,11]. However, the applicability and accuracy of these methods within the Indian context are not well studied, and few investigations have compared these estimates to the benchmark 24-hour urine collection [12]. Moreover, in India, previous studies on urinary salt excretion have focused exclusively on adults. To our knowledge, no study has objectively examined this important factor among adolescents using urine samples, although several studies have assessed dietary salt intake in this age group. Thus, this is the first study, to our knowledge, to objectively estimate urinary salt excretion among adolescents in the Indian context [13,14].

This study aimed to address current knowledge gaps by evaluating the 24-hour urinary excretory salt levels across age groups and comparing the gold standard of 24-hour urinary salt excretion with estimates from spot urine samples and 24-hour dietary recalls among adolescents and their parents in Chandigarh.

## Methods

This study was based on a cross-sectional survey carried out as part of a cluster randomized controlled trial to assess the impact of a school-based health promotion intervention package in reducing the behavioral risk factors associated with chronic diseases [15]. The survey was conducted among adolescents studying in public schools and one of their parents in the Union Territory of Chandigarh, India, from May 2018 to September 2019.

### Study Setting

The study was conducted in public schools as the Director of Public Instruction did not allow enrollment of private schools in the study. Data collected from the Department of School Education, Chandigarh, revealed that 106 public schools had eighth-grade classes, and on average, 10,790 adolescents studied in eighth grade in these schools. Most schools had 4 to 5 sections in the eighth-grade class. Each section of the class had about 30 adolescents.

### Participants

On the basis of the knowledge that behaviors contributing to chronic diseases, such as a preference for salty foods, often emerge during early adolescence, this study selected assenting eighth-grade adolescents and one of their parents based on their mutual preference [16]. Inclusion criteria required that participants planned to remain in the city for at least 1 year following recruitment and that the selected parent was younger than 65 years.

### Sample Size and Sampling

Details on the sample size calculation are described in the protocol paper of the broader cluster randomized controlled trial [17]. Broadly, calculations considered expected means or proportions for the control group, estimated intervention effects, and coefficient of variation values derived from adolescent data from previous studies [18-21]. To ensure sufficient power across all risk factors, the maximum calculated cluster size (n=12) was selected. Consequently, 12 clusters (schools), each with 30 adolescents, yielded an estimated total sample size of 360 adolescents and 360 parents.

### Data Collection Tools

Several tools were used for data collection after receiving assent from adolescents (Multimedia Appendix 1) and consent from parents (Multimedia Appendix 2). These tools included the interview schedule for recording sociodemographic information, behavioral risk factors, and anthropometric, physiological, and biochemical measures (Multimedia Appendix 3).

A 24-hour dietary recall questionnaire was used to assess the 24-hour dietary urinary excreted salt levels. Evidence suggests that a single 24-hour dietary recall is unable to provide the usual consumption pattern of the participants. To overcome this drawback, two 24-hour dietary recalls were collected for all participants on nonconsecutive days [22].

Twenty-four–hour urine and morning second-spot urine samples were collected to estimate the level of urinary salt excretion (g/day). To assess BMI, height was measured with a United Nations Children’s Fund standardized anthropometer to the nearest 0.1 cm, and weight was measured with a portable electronic weighing scale to the nearest 0.1 kg. Blood pressure (BP) was recorded for all participants using a digital sphygmomanometer (Omron Healthcare Co Ltd).

### Data Collection Methods

SK collected the dietary, anthropometric, and physiological data. Additionally, a team of 3 members, including SK and 2 field investigators, collected urine samples. The field investigators were qualified up to the graduation level, and they had received training for data collection related to urine samples.

Neither the investigator nor the data collectors were blinded to the intervention. Standard operating procedures were developed to maintain uniformity and standardization in sample collection and analysis (Multimedia Appendix 4)

After obtaining the study participants’ written informed consent, SK provided questionnaires to adolescents to record their responses in the classroom setting for recording sociodemographic characteristics and 24-hour dietary recall. SK was present in the classroom and explained each question to all the adolescents before they recorded their responses. Any query raised by the adolescents was answered.

To record the two 24-hour dietary recalls, all the adolescents in the same cluster filled out their forms themselves (self-administration). In comparison, in-person interviews were carried out with parents. The first 24-hour dietary recall was recorded during the first interaction with all the participants in the schools. The second 24-hour dietary recall was collected a week later. The data collection of the 24-hour dietary recall was manually recorded using the Multiple-Pass Method [22]. After collecting sociodemographic data and dietary recalls, participants’ anthropometric and BP measurements were obtained in a separate room one by one. An average of 3 readings was used for each participants’ BP measurement, with a resting period of at least 5 minutes between each reading. Finally, instructions for the collection of urine samples were provided. Specific days were fixed for collecting urine samples from the home setting. The details of BMI, BP, and 24-hour and second-spot urine samples collection are provided in MultimediaAppendices 4  5.

During Saturday home visits, participants received instructions regarding the process of 24-hour urine collection on Sundays and second-spot urine collection on Monday morning. Urine collection containers were provided to them. They were instructed to discard the first void of the morning and then start collecting the subsequent voids for the next 24 hours (Multimedia Appendix 6). Second-spot urine samples are preferred over first morning samples because they are less influenced by overnight concentration and better reflect daytime sodium excretion. This improves the accuracy of estimating 24-hour urinary sodium compared with first morning samples [23].

Participants were advised to write the time of their voids and whether they had collected each void in the collection container or not for 24 hours using a structured proforma (Multimedia Appendix 7). The urine samples were collected on Monday mornings and analyzed for urinary salt levels on the same day.

### Data Management and Analysis

The 24-hour dietary data were entered in a Microsoft Excel sheet. For the estimation of sodium intake, the Prospective Urban Rural Epidemiology study computer software was used [24]. This tool was developed explicitly for assessing the intake of Indian foods and has been previously validated in Indian settings [24]. The details of the estimation of 24-hour urinary excreted salt levels using this tool have been provided in Multimedia Appendix 8. Twenty-four–hour dietary urinary excreted salt levels from the 2 recalls of every participant were calculated separately and then averaged and multiplied by a correction factor of 0.9 to estimate their usual 24-hour urinary excreted salt levels.

For BMI categorization of adolescents into severe thinness, thinness, normal weight, overweight, and obese categories, WHO growth reference percentiles that were age and sex specific for the 5- to 19-year age group were used [25]. For parents, WHO Asia-specific BMI cutoffs were used to categorize them into underweight, normal weight, overweight, and obese categories [26].

For BP assessment among adolescents, percentiles for systolic BP and diastolic BP were assessed based on each adolescent’s height, age, and sex (normal: <90th percentile; borderline-elevated BP: systolic BP or diastolic BP ≥90th percentile but <95th percentile; elevated BP: systolic BP or diastolic BP ≥95th percentile). Recent 2025 American College of Cardiology and American Heart Association guidelines were used to categorize adult participants into normal, elevated, stage 1, and stage 2 hypertension [27].

Urinary excreted salt levels were estimated from spot urine samples using a series of established estimation equations. The equations used were Tanaka, Kawasaki, and INTERSALT with potassium [28] (Multimedia Appendix 9). Twenty-four–hour urinary excreted salt levels values that were greater than 3 SDs above or below the mean were excluded from all analyses. Twenty-four–hour urinary salt excretion was estimated from 24-hour urine samples using the formula mentioned in Multimedia Appendix 9. As both 24-hour urine collections (gold standard) and spot urine–based equations estimate 24-hour urinary salt excretion, the salt intake derived from 24-hour dietary recall was adjusted using a 0.9 correction factor to approximate urinary excreted salt. This adjustment is based on the established physiological evidence that approximately 90% of ingested salt is excreted in urine under steady-state conditions [29]. Paired samples 2-tailed *t* tests were used to compare mean population 24-hour urinary excreted salt levels estimated by the 24-hour urine collections, spot urine equation-based methods, and 24-hour dietary recalls. To determine the agreement between individual-level 24-hour urinary excreted salt levels estimated from 24-hour collections and methods based on spot urine collections and dietary recall, Bland-Altman plots were used. The difference between the methods was plotted against the mean of the 2 methods. Data are presented as mean (95% CI) unless otherwise specified. Statistical analyses were conducted using R (version 4.3.0; R Foundation for Statistical Computing).

### Ethical Considerations

Ethics approval was obtained from the institutional ethics committee of the Post-Graduate Institute of Medical Education and Research, Chandigarh (INT/IEC/2018/000082; January 22, 2019). Permission to conduct the study in schools was granted by the Department of Education, Chandigarh, with letter number 1296-DSE-UT-S5-11(65)11-II. As this study was part of a cluster randomized controlled trial, the study was registered in Clinical Trials Registry-India (CTRI/2019/09/021452).

Written assent of adolescents and written consent from their parents were obtained after informing them about the study purpose, data to be collected, estimated time for data collection, the confidentiality of the data, and risks involved before their enrollment in the study (MultimediaAppendices 1  2).

## Results

### Participants

**Figure 1.**
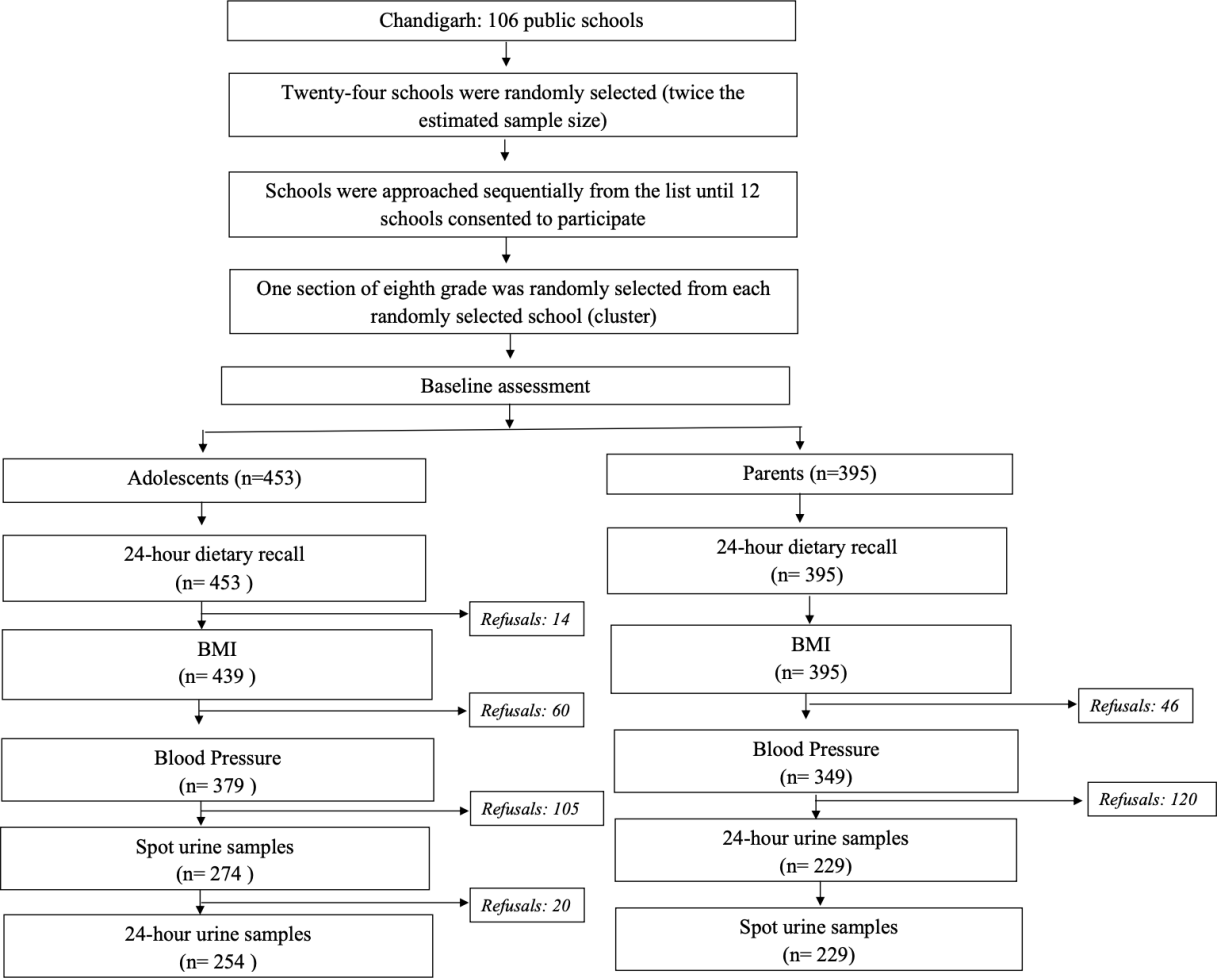
Sampling strategy and participation rates for various indicators among all study participants.

Of the 462 eligible adolescents, 453 (98.1%) consented to be part of the study (Figure 1). One parent was supposed to be enrolled in the study for each adolescent. However, 395 consenting parents enrolled in the study.

The mean age was 13 (SD 0.4) years for adolescents and 37 (SD 0.3) years for parents (Table 1). Of all participating adolescents, 46.1% (209/453) were girls, and of all parents, 61% (241/395) were mothers. Nearly a quarter (105/439, 23.9%) of the participating adolescents were underweight, whereas half (197/395, 49.9%) of the parents were either overweight or obese (Table 1). Among adolescents, 3.4% (13/379) had stage 2 hypertension compared with 25.5% (89/349) of parents. There were differential participation rates for each of the indicators used in this study, as shown in Figure 1.

**Table 1.** Study participants’ characteristics.

Participant characteristics	Adolescents	Parents
Age (y), mean (SD; 95% CI)	13.12 (0.4; 13.05-13.19)	37.1 (0.3; 36.54-37.67)
Female, n (%)	209/453 (46.1)	241/395 (61.0)
Weight (kg), mean (SD; 95% CI)	39.4 (0.45; 38.6-40.3)	61.95 (0.63; 60.71-63.19)
BMI (kg/m^2^), mean (SD; 95% CI)	17.4 (0.17; 17.1-17.8)	25.11 (0.22; 24.67-25.55)
BMI classification^a^^b^, n (%)		
Underweight	105/439 (23.9)	27/395 (6.8)
Overweight and obese	46/439 (10.5)	197/395 (49.9)
Blood pressure^c^^,^^d^, n (%)		
Normal	346/379 (91.3)	204/349 (53.7)
Hypertension stage 1	17/379 (4.5)	56/349 (14.7)
Hypertension stage 2	13/379 (3.4)	89/349 (23.4)

a Adolescent BMI categories were based on World Health Organization growth reference percentiles age and sex specific for 5-19 y (2007): ≤−2 SD, severe thinness; >−2 SD to ≤−1 SD, thinness; >−1 SD to ≤+1 SD, normal weight; >+1 SD to ≤+2 SD, overweight; and >+2 SD, obesity.

b Parent BMI categories were defined as follows: underweight, <18.5 kg/m2; normal weight, 18.5-24.9 kg/m2; overweight, 25.0-29.9 kg/m2; and obese, ≥30.0 kg/m2.

c Adolescent blood pressure (BP) categories were based on age-, height-, and sex-specific percentiles: normal, <90th percentile; borderline-elevated BP, systolic BP or diastolic BP ≥90th percentile but <95th percentile; elevated BP, systolic BP or diastolic BP ≥95th percentile.

d Parent BP categories were defined as follows: normal, systolic BP <120 mm Hg and diastolic BP <80 mm Hg; elevated BP, systolic BP 120-129 mm Hg and diastolic BP <80 mm Hg; stage 1 hypertension, systolic BP 130-139 mm Hg or diastolic BP 80-89 mm Hg; and stage 2 hypertension, systolic BP ≥140 mm Hg or diastolic BP ≥90 mm Hg.

### Urinary Excreted Salt Levels

Based on the 24-hour urine samples, the measured 24-hour urinary excreted salt level among adolescents was 6.95 g/day (SD 0.24; 95% CI 6.48-7.42), and for their parents it was 7.73 g/day (SD 0.3; 95% CI 7.18-8.28). These measured estimates were compared with estimated 24-hour urinary excreted salt levels derived from various urine equations by using spot urine samples and 24-hour dietary recall. (Multimedia Appendix 9).

Twenty-four–hour dietary recall estimates of 24-hour urinary excreted salt levels were the lowest for both adolescents (mean 4.15, SD 0.08 g/day; 95% CI 3.99-4.31) and parents (mean 3.62, SD 0.08 g/day; 95% CI 3.47, 3.77; Table 2).

**Table 2.** Population-level estimates of 24-hour urinary excreted salt among adolescents and their parents^a^.

	Adolescents (g/day), mean (SD; 95% CI)	Parents (g/day), mean (SD; 95% CI)	
Measured 24-hour urinary salt	6.95 (0.24; 6.48-7.42)	7.73 (0.3; 7.18-8.28)	
Estimated 24-hour dietary salt	4.15 (0.08; 3.99-4.31)	3.62 (0.08; 3.47-3.77)	
Estimated Kawasaki equation	12.83 (0.5; 11.92-13.74)	12.72 (0.3; 12.17-13.28)	
Estimated Tanaka equation	5.79 (0.13; 5.55-6.04)	6.89 (0.12; 6.65-7.13)	
Estimated INTERSALT equation with potassium	7.97 (0.4; 7.16-8.77)	8.12 (0.4; 7.36-8.95)	

a Sample sizes varied by indicator and are presented in Figure 1. Equations used to estimate urinary salt excretion are provided in Multimedia Appendix 9.

### Agreement Between Methods of Estimating 24-Hour Urinary Excreted Salt Levels

**Figure 2.**
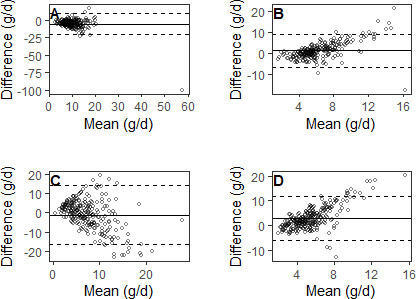
Bland-Altman plots comparing 24-hour urinary excretory salt estimates from 24-hour urine, spot urine samples, and 24-hour dietary recall in adolescents. Bias and 95% limits of agreement (LoA) in comparing 24-hour urinary salt excretion assessed through 24-hour urine samples (gold standard) with (A) Kawasaki (bias −5.78, LoA −21.13 to 9.57), (B) Tanaka (bias 1.29, LoA −6.52 to 9.10), (C) INTERSALT with potassium (bias −1.16, LoA −16.30 to 13.99), and (D) 24-hour dietary recall (bias 2.92, LoA −6.00 to 11.85).

For population-level 24-hour urinary excreted salt level estimation, comparisons among methods revealed that among adolescents there were no significant differences between the estimates derived from 24-hour urine collections and those obtained using the Kawasaki (*P*<0.001) and Tanaka equations (*P*=0.008), as well as 24-hour dietary recall (*P*<0.001). The INTERSALT equation with potassium slightly underestimated the 24-hour salt levels (mean −1.16 g/day; 95% limits of agreement −16.3 to 13.99 g/day; *P*=.02; Figure 2).

**Figure 3.**
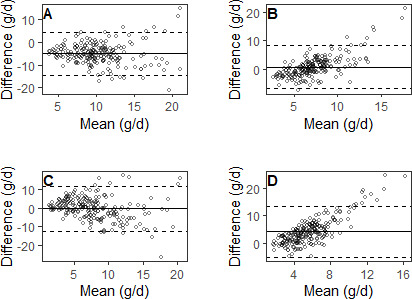
Bland-Altman plots comparing 24-hour urinary excretory salt estimates from 24-hour urine samples, spot urine samples, and 24-hour dietary recall in parents. The graphs show bias and 95% limits of agreement (LoA) in assessing 24-hour urinary salt excretion through 24-hour urine samples (the gold standard) with (A) Kawasaki (bias −5.16, LoA −14.42 to 4.10), (B) Tanaka (bias 0.71, LoA −6.82 to 8.24), (C) INTERSALT with potassium (bias −0.23, LoA −12.22 to 11.75), and (D) 24-hour dietary recall (bias 4.18, LoA −5.25 to 13.60) methods.

Among parents, there were no significant differences between the estimates derived from 24-hour urine collections and those obtained using the Kawasaki and INTERSALT equations, as well as 24-hour dietary recall. Tanaka slightly overestimated the 24-hour salt levels (mean 0.71 g/day; 95% limits of agreement −6.82 to 8.24 g/day; *P*=.008; Figure 3).

## Discussion

### Key Findings

This study suggests that spot urine samples can be practical alternatives to 24-hour collections for estimating population-level 24-hour urinary excreted salt levels in Indian populations, with INTERSALT with potassium being the most suitable method. The INTERSALT with potassium equation showed closer agreement with the gold standard, 24-hour urine samples, at the population level; in contrast, the 24-hour dietary recall method tended to underestimate the urinary excreted salt levels. Adolescence is a period of significant physiological change with distinct nutritional requirements; therefore, 24-hour urine samples remain the gold standard for assessing 24-hour urinary excreted salt levels in this age group. However, understanding 24-hour urinary excreted salt levels in this group is crucial, as studies suggest that high salt consumption in early years is a major risk factor for hypertension later in life. Importantly, this is a modifiable behavior that can be addressed during early adolescence through appropriate interventions [5,9].

The mean population intake estimated in the study is comparable to the earlier studies; for instance, standard 24-hour urinary salt excretion, widely regarded as the gold standard, was measured at 7 g/day among adolescents and 8 g/day among their parents [13,30]. These findings align closely with previous studies, such as one conducted in China, where the average 24-hour urinary salt excretion for adolescents was 8.2 g/day using 24-hour urine samples [31]. Additionally, an Indian study estimated the weighted mean 24-hour urinary excreted salt levels from 24-hour urine samples to be 8.59 g/day in Delhi and 9.46 g/day in Andhra Pradesh, further supporting the validity of our findings [30]. In this study, the mean 24-hour urinary salt excretion estimated through the INTERSALT equation with potassium, based on spot urine samples, was 8.0 g/day for adolescents and 8.12 g/day for their parents. These values are consistent with Dong et al [31], where adolescent 24-hour urinary salt excretion was reported as 8.4 g/day using the same equation. Another Indian study using the INTERSALT equation with potassium yielded comparable results, with estimates of 8.13 g/day in Delhi and 8.81 g/day in Andhra Pradesh [30]. Together, these findings suggest that the INTERSALT equation with potassium can yield population-level estimates closely aligned with those obtained from 24-hour urine collections for adults, making spot urine samples a feasible option in settings where 24-hour collections are challenging.

The biochemical assessment of 24-hour urinary excreted salt levels, based on urinary measures, proves to be more accurate than dietary recall methods. In this study, dietary recalls on 2 nonconsecutive days estimated 24-hour urinary excreted salt levels between 4 and 4.5 g/day for participants across all age groups. This underestimation is supported by the literature; for example, McLean et al [32] conducted a meta-analysis of 28 studies and confirmed that dietary recall methods consistently underestimate 24-hour urinary excreted salt levels when compared with 24-hour urine collections.

Among the various methods for estimating 24-hour urinary excreted salt levels, the INTERSALT equation with potassium applied to spot urine samples produced no statistical difference from the gold standard of 24-hour urine collection, with differences of 0.23 g/day among parents. Previous studies indicate that among urine equations, including Kawasaki, Tanaka, and INTERSALT, the INTERSALT method demonstrates the most reliable performance in estimating mean population sodium intake, minimizing bias effectively [31,33]. However, there was a significant difference between 24-hour salt levels assessed through INTERSALT with potassium for adolescents. This variation may be attributed to differences in urinary potassium levels between adolescents and adults, influenced by growth-related physiological changes, dietary habits, and hormonal fluctuations during adolescence, all of which can affect potassium excretion [34].

### Strengths and Limitations

This study has several strengths and limitations. Pilot testing was conducted prior to data collection to assess feasibility and refine research instruments, helping to address potential challenges. A key strength is the use of validated tools in the Indian context, along with standardized equipment and protocols for BP assessment.

The 24-hour dietary recall is a reliable method for assessing population-level intake compared with tools such as the Food Frequency Questionnaire. Although it may not fully capture usual intake, we used recalls on 2 nonconsecutive days to improve reliability. The study was adequately powered, and the findings can inform sample size calculations for future research.

A limitation is the exclusion of private schools, which may affect representation across socioeconomic groups. However, as a large proportion of the Indian population belongs to low- and middle-income groups, the findings remain reasonably generalizable to the Indian settings.

Overall, this study supports the utility of spot urine samples, specifically with equations such as the INTERSALT equation with potassium, for accurately estimating population-level 24-hour urinary excreted salt levels in adults.

### Conclusions

In settings where 24-hour urine collections are impractical, spot urine equations such as INTERSALT with potassium offer effective alternatives for estimating 24-hour urinary sodium excretion in adult populations in the Indian context. However, 24-hour dietary recall methods often underestimate 24-hour urinary excreted salt levels, and their accuracy can be significantly improved when combined with objective measurements. This combined approach is especially valuable for designing and implementing effective salt reduction interventions. As part of a larger cluster randomized controlled trial, the findings can support objective assessment of urinary salt excretion in resource-limited settings. Future interventional studies may also adopt the tools used in this study to monitor changes in behavioral risk factors, including dietary behaviors assessed through 24-hour recall.

## Supplementary material

10.2196/85549 Multimedia Appendix 1 Participant informed assent form.

10.2196/85549 Multimedia Appendix 2 Parental informed consent form.

10.2196/85549 Multimedia Appendix 3 Data collection tool.

10.2196/85549 Multimedia Appendix 4 Standard operating procedure for urine samples (24-hour and spot).

10.2196/85549 Multimedia Appendix 5 Standardized tools and methods to record height, weight, and blood pressure.

10.2196/85549 Multimedia Appendix 6 Approaches of 24-hour and spot urine sample collection.

10.2196/85549 Multimedia Appendix 7 Twenty-four-hour urine collection: field form.

10.2196/85549 Multimedia Appendix 8 Estimation of dietary salt.

10.2196/85549 Multimedia Appendix 9 Estimation of urinary salt.

## Abbreviations

BP: blood pressure
WHO: World Health Organization
