# Supplementary material for: Comparison of Measured 24-Hour Urinary Salt Excretion With Spot Urine and 24-Hour Dietary Recall Estimates Among Adolescents and Parents: Cross-Sectional Study
Source: JMIR Public Health Surveill. 2026 Jun 30;12:e85549. doi: 10.2196/85549 (PMC13317844; doi:10.2196/85549)
Supplement: Multimedia Appendix 1 [file publichealth-v12-e85549-s001.pdf]

APPENDIX S1: Participant Informed Assent Form

Project Title: Health Promotion Intervention Package for Prevention of Underlying Behavioral Risk Factors of Chronic Diseases: A Cluster Randomized Controlled Trial among Adolescents in School Settings

Principal Investigator: Ms. Sandeep Kaur

We are doing a research study to promote healthy behavioral practices among school going children, their parents & teachers to prevent risk factors of various chronic diseases. If you decide that you want to be part of this study, you will be asked to answer some health behavior related questions pertaining to dietary intake, physical activity, alcohol & tobacco use, etc .Your physical and anthropometric measurements will also be taken. We think this will take you around 30-45 minutes. Your blood pressure and blood glucose level will be measured.

No physical as well as psychological risks are involved in the present study.

Everyone who takes part in this study will benefit. A benefit means that something good happens to you. We think that the benefit for you will be that you will be get knowledge and learn the ways in which you can maintain your health and improve it by adapting to healthier behavioral practices and hence reducing the risk of getting these lifestyle diseases in future and in their maintenance, if already suffering from any.

When we are finished with this study we will write a report about what was learned. This report will not include your name or that you were in the study.

You do not have to be in this study if you do not want to be. You can stop participating at any time of the study. Your parents know about the study too.

If you decide you want to be in this study, please sign your name.

I, ________________________________, want to be in this research study.

___________________________________ _________

(Sign your name here) (Date)
