# Supplementary material for: Comparison of Measured 24-Hour Urinary Salt Excretion With Spot Urine and 24-Hour Dietary Recall Estimates Among Adolescents and Parents: Cross-Sectional Study
Source: JMIR Public Health Surveill. 2026 Jun 30;12:e85549. doi: 10.2196/85549 (PMC13317844; doi:10.2196/85549)
Supplement: Multimedia Appendix 2 [file publichealth-v12-e85549-s002.pdf]

APPENDIX S2: Parental Informed Consent Form

Project title

Health Promotion Intervention Package for Prevention of Underlying Behavioral Risk Factors of Chronic Diseases: A Cluster Randomized Controlled Trial among Adolescents in School Settings

Introduction

Your child and you have been invited to join a research study entitled, 'Health Promotion Intervention Package for Prevention of Underlying Behavioral Risk Factors of Chronic Diseases: A Cluster Randomized Controlled Trial among Adolescents in School Settings' by Ms. Sandeep Kaur, PhD Scholar, School of Public Health, PGIMER, Chandigarh. Please take whatever time you need to discuss the study with your family and friends, or anyone else you wish to. The decision to let you and your child join, or not to join is up to you. In this research study, we are providing health promotion intervention to promote healthy behavioral practices among school going children, their parents & teachers to prevent the risk factors of various chronic diseases

What is involved in the study?

Your child and you will be asked to answer some health behavior related questions pertaining to dietary intake, physical activity, tobacco & alcohol use, etc. His/her and your physical and anthropometric measurements will also be taken. We think this will take him/her and you around 30-45 minutes each. Your child and yours blood pressure and blood glucose level will be measured. Your child can stop participating at any time. If your child stops he/she will not lose any benefits.

Risks

This study does not involve any physical and psychological risks.

Benefits of being a part of this study?

It is reasonable to expect the following benefits from this research: Benefit is that you and your child will get knowledge and learn the ways in which you can maintain your health and improve it by adapting to healthier behavioral practices and hence reducing the risk of getting these behavioral diseases in future and their maintenance, if already suffering from any.

Others may benefit in the future from the information we find in this study.

Confidentiality

Your child's and your name will not be used when data from this study are published but a code (a number) would be used purely for the purpose of monitoring the data collection process and will not be revealed beyond the study team. Every effort will be made to keep clinical records, research records, and other personal information confidential.

Your child rights as a research participant?

Participation in this study is voluntary. Your child has the right not to participate at all or to leave the study at any time. Deciding not to participate or choosing to leave the study will not result in any penalty or loss

of benefits to which your child is entitled, and it will not harm his/her relationship with his/ her school authorities.

Permission for a Child to Participate in Research

As parent or legal guardian, I authorize __________________________________ (adolescent's name) to become a participant in the research study described in this form.

Adolescent's Date of Birth

Parent or Legal Guardian's Signature Date

Consent to be a participant of the study

If you decide you want to be in this study, please sign your name.

I, __________________________________, want to be in this research study.
