# Supplementary material for: Comparison of Measured 24-Hour Urinary Salt Excretion With Spot Urine and 24-Hour Dietary Recall Estimates Among Adolescents and Parents: Cross-Sectional Study
Source: JMIR Public Health Surveill. 2026 Jun 30;12:e85549. doi: 10.2196/85549 (PMC13317844; doi:10.2196/85549)
Supplement: Multimedia Appendix 3 [file publichealth-v12-e85549-s003.pdf]

APPENDIX S3: Data Collection Tool

Part 1: Information (Adolescents)

Student's ID	**Caste** (1=General, 2=Other backward class 3=Scheduled caste, 4=Scheduled tribe)
**Age (in years):**	**Area of residence:** 1= urban, 2= urban slum<br>3=Rural
**Sex: Male/Female**	**Address:**
**Religion (Hindu=1, Sikh=2, Muslim=3,**<br>**Christian =4, Others (Jain, Buddhist)=5)**	**Mobile number:**

1. **Please provide the following details about your family members?**

**S. No**	**Earning family member**			**Education** (1=Illiterate, 2=primary, 3= Middle, 4=Matric, 5=senior secondary, 6= Graduation and above)	**Occupation** (1=Housewife, 2= Skilled worker, 3=Non-skilled worker, 4=Business/shop owner; 5= Professional, 6=Un-employed, 7= Pensioner, 8=others (specify))	**Income** (Indian Rupees)
	**Name**	**Relationship**	**Age (in years)**			
1						
2						
3						
4						

2. **Self-assessed health:** Which of the following best describes your general heal

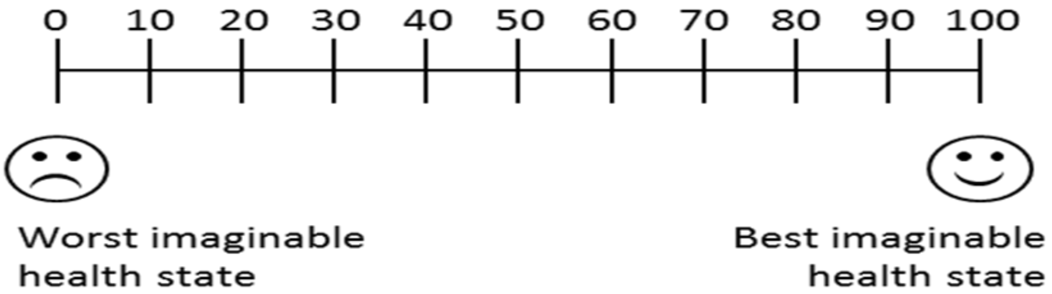

Part 2: 24 hour dietary recall

Time	Occasion*	What and how much did you have? (Food, drinks, etc.)

Occasion: 1: breakfast, 2: brunch and evening snacks, 3: lunch, 4: dinner, 5: late night meal, 6: fruit, 7: food & beverage break, 8: any other (specify)
