# Supplementary material for: Comparison of Measured 24-Hour Urinary Salt Excretion With Spot Urine and 24-Hour Dietary Recall Estimates Among Adolescents and Parents: Cross-Sectional Study
Source: JMIR Public Health Surveill. 2026 Jun 30;12:e85549. doi: 10.2196/85549 (PMC13317844; doi:10.2196/85549)
Supplement: Multimedia Appendix 4 [file publichealth-v12-e85549-s004.pdf]

APPENDIX S4: Standard Operating Procedure for Urine Samples (24-Hour And Spot)

Introduction: A 24-hour collection of urine into a plain container can be used to estimate excretion of sodium.

Purpose: The SOP provides instructions for specimen collection, handling and labeling to promote uniformity.

Policy: All specimens must be labeled with the participants name and a special six digit code already assigned to each participant. For parents 8 digit codes that include age will be used.

Equipment (for one participant)

- Participant information sheet
- One 3-litre 24hr urine collection container. No additives.
- Jug
- two 50 ml container for final sample collection of 24-hour urine sample and spot urine sample

Procedure: Pre-preparation

Labeling Specimens and Containers

- Information must be placed on a label which is on the container.
- All specimens MUST be labeled with the participants name and a special six digit code already assigned to each participant. For parents 8 digit codes that include age will be used.
- Identifying information placed on covers of containers is NOT acceptable.

Outside Contamination of Specimen Container

- The outside of containers or accompanying requisition slips should not be contaminated, (i.e. contamination of a container with feces, urine, etc.)
- If the specimen container is inadvertently contaminated at the time of collection and it is not feasible to recollect a specimen, the contaminated container should be placed inside a plastic bag and sealed, and this fact should be noted on the requisition slip.
- Contaminated requisition slips should be discarded and a new one filled out.

Damaged Specimen Container

- Glass or plastic containers which are cracked or broken so that the specimen may leak out (or may be considered a hazard to those handling the object) should not be submitted for examination. Dispose in the appropriate rigid, biohazard container.
- Specimens must be delivered promptly to the laboratory after collection.

Steps for sample collection

- Each participant will be visited by a research team member.

- It is important that the participant understand the process, and to that end, a full verbal explanation should be given along with a written information sheet repeating the same details. This sheet should have a contact name and phone number included, allowing the patient to call and ask any questions or clarify any doubts.
- Participants will be given schedule sheet where they will note down the time each time they pass urine and will also note time and mention if they fail to collect any sample during the 24 hour time.
- Ensure that the participant understands that even a single sample collection missed will impair/invalidate the results obtained from that collection.

Following instructions to be shared with participants for 24-Hour Urine Collection

- Instruct the participant to avoid alcoholic beverages, vitamins, and other medication (if possible) after they have been provided with the containers for sample collection till the time the samples are collected back from them.
- Instruct the participant to check with his/her physician prior to discontinuing any medications.
- Instruct the participant to not exceed his/her normal intake of liquids during the day before and the day of collection unless his/her physician gives the participant specific directions to do otherwise.
- Instruct the participant to keep the specimen in cool & dark place during the 24 hour collection period.
- The 24-hour collection period begins when the participant gets up in the morning and empties his/her bladder. DO NOT COLLECT THIS URINE! But do record the date and time of this voiding on the Sheet provided. Be sure to collect ALL urine (Day and Night) for the next 24 hours.
- Instruct the participant to make his/her final collection when he/she empties his/her bladder the next morning, approximately 24 hours from the time marked on his/her specimen container.

Sample collection technique

The 750 participants enrolled in the study will be categorized into 4 clusters based on their residential addresses.

Four field investigators will adopt 15 households each. They will explain the purpose of the study and process of the test to the participants on Saturday and will give them containers for 24 hour urine collection. Participants will start the urine collection on Sunday morning till Monday morning. Each field worker will contact to each of their adopted households through phone (1st call on Sunday morning whenever the participants wakes, 2nd call at or around 1PM on Sunday and 3rd call at 6:30 AM on Monday morning) to give participants reminder, to clear query of participants, if any.

On Monday morning the field investigators will note down the urine volume and take the 24 hour urine samples and will also collect spot urine and will transport both the samples (24 hour and spot urine) to the laboratory for further analysis. It is expected that each week 60 households (120 samples) will be covered. This process will be repeated for six weeks (on Saturday, Sunday & Monday) to collect all 750 samples.

Standardized laboratory methods to assess excretory urinary salt
All urine samples were analyzes in Beckman Coulter AU 5800 analyzer (2020)

Sodium

Principle

ISE Electrolyte Buffer reagent and ISE Electrolyte Reference reagent, when used in conjunction with UniCel DxC 600/800 System(s) and SYNCHRON Systems AQUA CAL 1, 2 and 3, are intended for the quantitative determination of sodiumconcentration in human serum, plasma or urine.

Methodology

The SYNCHRON System(s) determines sodium ion concentration by indirect potentiometry utilizing two glass sodium electrodes (one acts as the reference electrode). To measure sodium concentrations, a precise volume of sample (40 microliters) is mixed with a buffered solution. The ratio used is one part sample to 33 parts buffer. The high molar strength buffer is used to establish a constant activity coefficient for sodium ions, calibrating the electrode to concentration values.

Chemical Reaction Scheme

The sodium electrode is made of lithium-sodium-aluminum-silicate glass. It is essential that the outer layer of the glass electrode is adequately hydrated. When the sample buffer mixture contacts the electrode, sodium ions in the sample undergo an ion exchange process with the sodium ions in the hydrated layer of the electrode. Changes in electrode potential occur as the ion exchange process takes place. These changes in electrode potential are referenced to the reference electrode. The "referenced potential" follows the Nernst equation and allows the calculation of sodium concentration in the sample: For more accurate measurement, the reference reagent containing sodium ions is introduced into the flow cell after the sample cycle, and the same ion exchange process takes place. The differential potential (voltage) between sample and reference reagent cycles is used for the calculation. Under ideal conditions, the electrode imparts a selectivity of 300:1 over potassium and is insensitive to hydrogen ions in solutions buffered from pH 6 to 10.

$$E = \text{Constant} + (\text{slope})\,(\log[Na^+])$$

E015247L.EPS
