# Supplementary material for: Comparison of Measured 24-Hour Urinary Salt Excretion With Spot Urine and 24-Hour Dietary Recall Estimates Among Adolescents and Parents: Cross-Sectional Study
Source: JMIR Public Health Surveill. 2026 Jun 30;12:e85549. doi: 10.2196/85549 (PMC13317844; doi:10.2196/85549)
Supplement: Multimedia Appendix 5 [file publichealth-v12-e85549-s005.pdf]

APPENDIX S5: Standardized Tools and Methods to Record Height, Weight and Blood Pressure

Height: For height measurement, participants were asked to stand straight with their feet bare to the vertical background of the UNICEF standardized height board placed on the flat surface. The heels of the participants were joined together with toes pointing outward at about 60 degrees. In case of any high hairstyle, the participant was requested to remove it. Three points, namely, buttocks, scapulae and head of the participants, were in contact with the vertical backboard. The arms of participants were placed straight and freely at the sides with the palm of their hands facing thighs. The participant's head was adjusted to be in Frankfort Horizontal Plane position. Once the participant was correctly positioned on the height board, the horizontal bar was pulled down until it touched the head's top. The bar was locked in place, and the height was recorded to the nearest 0.1 cm.

Weight: Participants were asked to remove heavy clothes (jackets, trousers, coats and shirts, etc.) and shoes for recording weight. The participants were requested to stand at the centre part of the digital weighing scale with their weight distributed evenly on both feet. The weight was recorded to the nearest 0.1 kg. The portable digital weighing scale was calibrated after every 20th reading.

Blood Pressure: For blood pressure, an average of three readings was recorded for all participants using a digital sphygmomanometer (Omron). The instrument was placed on a level with the participant's heart while recording the reading. The participant's arm was positioned correctly with the bottom edge of the cuff 1-2 cm above the elbow and the marker placed in the centre of the inner arm and the cuff snugly fitted on the arm. Each participant's blood pressure was taken three times with a one-minute gap between each reading.
