# Supplementary figures and images for: Comparison of Measured 24-Hour Urinary Salt Excretion With Spot Urine and 24-Hour Dietary Recall Estimates Among Adolescents and Parents: Cross-Sectional Study
Source: JMIR Public Health Surveill. 2026 Jun 30;12:e85549. doi: 10.2196/85549 (PMC13317844; doi:10.2196/85549)

## APPENDIX S6: Approaches of 24-Hour and Spot Urine Sample Collection

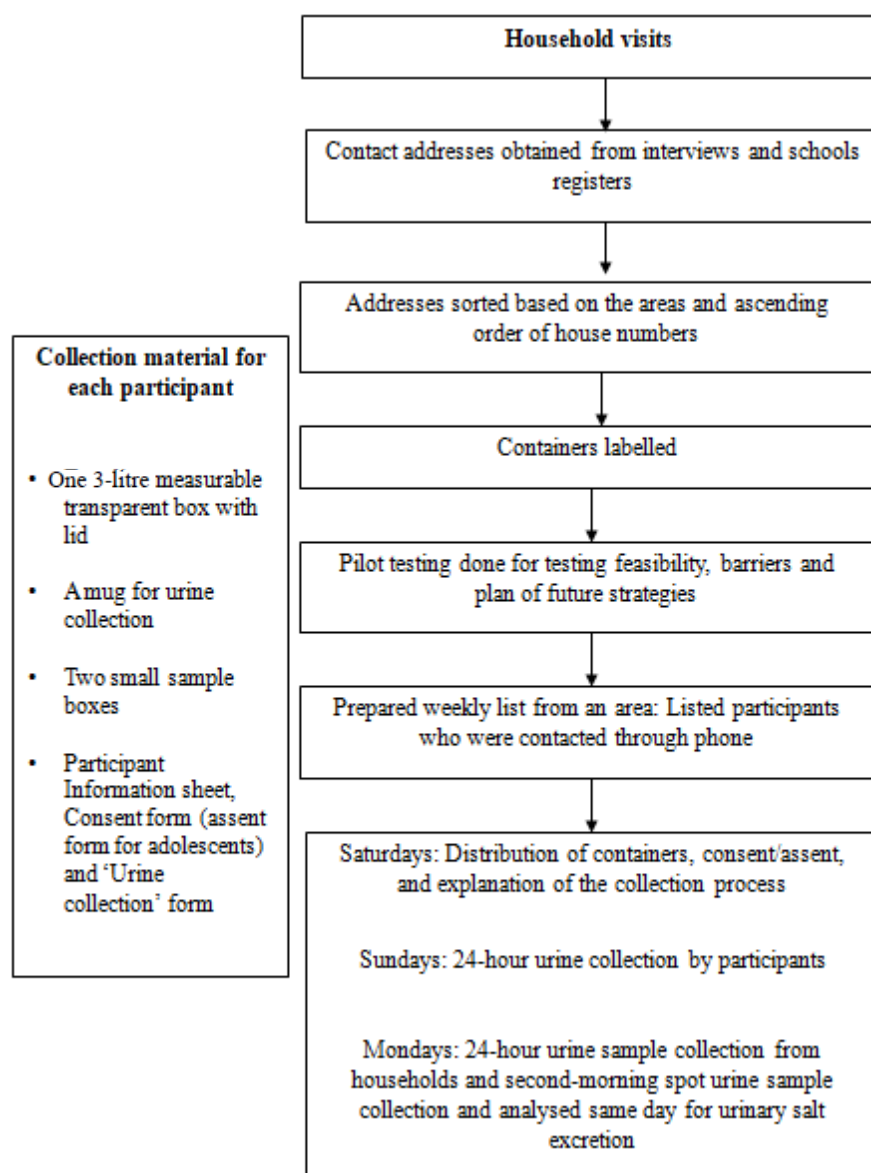

Supplement: Multimedia Appendix 6 [file publichealth-v12-e85549-s006.pdf]
