# Supplementary material for: Comparison of Measured 24-Hour Urinary Salt Excretion With Spot Urine and 24-Hour Dietary Recall Estimates Among Adolescents and Parents: Cross-Sectional Study
Source: JMIR Public Health Surveill. 2026 Jun 30;12:e85549. doi: 10.2196/85549 (PMC13317844; doi:10.2196/85549)
Supplement: Multimedia Appendix 7 [file publichealth-v12-e85549-s007.pdf]

APPENDIX S7: 24-Hour Urine Collection: Field-Form

Please fill the following information:

Name:

Age:

Code:

Collection	Start Date	Start Time	Collected/Missed
Collection 1			
Collection 2			
Collection 3			
Collection 4			
Collection 5			
Collection 6			
Collection	Stop Date	Stop Time	
