# Supplementary material for: Comparison of Measured 24-Hour Urinary Salt Excretion With Spot Urine and 24-Hour Dietary Recall Estimates Among Adolescents and Parents: Cross-Sectional Study
Source: JMIR Public Health Surveill. 2026 Jun 30;12:e85549. doi: 10.2196/85549 (PMC13317844; doi:10.2196/85549)
Supplement: Multimedia Appendix 8 [file publichealth-v12-e85549-s008.pdf]

## APPENDIX S8: Estimation of Dietary Salt

**List of food items in pure study software**

| S. No. | Items included in fruits estimation g/d | Items included in vegetable estimation g/d | Items included in sugar estimation g/d | Other additional food items for calculating macro, micro-nutrients, vitamins and minerals | |
|---|---|---|---|---|---|
| | a | b | c | d | (a+b+c+d) |
| 1 | Banana | Ghia | Cornflakes | Chapati with Ghee | Egg gravy |
| 2 | Mango | Tori | Boiled Rice | Chapati dry | Chicken Gravy |
| 3 | Apple | Karela | Dhalia, all types | Bread | Chicken fried / roasted etc. |
| 4 | Water melon | Ladies finger (Bhindi) | Rusk | Toast | Mutton, pork, beef curries. |
| 5 | Peaches | Yam | Pasta | Bread rolls | Fish gravy |
| 6 | Pears | Capsicum | Biscuits, salted | Buns | Fish fried. |
| 7 | Orange | Tinda | Biscuits sweet, cream etc. | Pulao / fried rice / zeera rice | Ham, salami, bacon etc. |
| 8 | Gauva | Saag | Chips, Khichre etc. | Plain Parantha | Bhel Puri |
| 9 | Papaya | Methi/Methey | Ice cream | Stuffed Parantha | Chats |
| 10 | Plum | Spinach | Cakes | Porridge | Patties |
| 11 | Grapes | Cabbage | Pastries | Puri | Pakoda |
| 12 | Musambi | Cauliflower | Custard | Bhature | Samosas |
| 13 | Pineapple | Brinjal | Kheer | Uppama | Mathies (NAMKEEN) |
| 14 | Pomegranete | Drumstick | Gulab Jamun | Poha | Namkeen, mixture etc. |
| 15 | Zizyphus | Colocasia | Jalebi | Noodles | Groundnuts |
| 16 | | Fresh peas | Rasgulla | Macaroni | Cashew nuts |
| 17 | | Kathal | Rasmalai | Pizza | Raitha with boondi |
| 18 | | Beans | Sweet Mathi | Burger | Raitha with vegetables |
| 19 | | | Malpuda | Dosa | Namkeen lassi |
| 20 | | | Halwa | Idli | Whisky |
| 21 | | | Carrrot Halwa | Khichidi | Beer |
| 22 | | | Ladoo | Rajma | Wine |
| 23 | | | Pinni | Saboot Moong | Cheese |
| 24 | | | Gujia | Whole gram curries-Black | Butter / Cream |

| S. No. | Items included in fruits estimation g/d | Items included in vegetable estimation g/d | Items included in sugar estimation g/d | Other additional food items for calculating macro, micro-nutrients, vitamins and minerals | |
|---|---|---|---|---|---|
| | a | b | c | d | (a+b+c+d) |
| 25 | | | Chocolates | Whole gram curries-White | Ghee |
| 26 | | | Candies | Dehusked dhal, all types | Added salt |
| 27 | | | Milk | Dhals with husk, all types | |
| 28 | | | Flavoured milk | Buttermilk curry | |
| 29 | | | TEA | Kofta curry | |
| 30 | | | Coffee | Green leafy vegetable curries | |
| 31 | | | Curd | Paneer gravy | |
| 32 | | | Sweet lassi | Mint / coriander chutney | |
| 33 | | | Fresh fruit juices | Tomato, tamrind, other chutneys | |
| 34 | | | Fruit juices packed | Veg/ Non veg soup | |
| 35 | | | Lemon water | Salad with raw vegetables | |
| 36 | | | Fanta, pepsi etc. | salad with sprouted grams | |
| 37 | | | Tomato Sauce | Papad Roasted | |
| 38 | | | Added sugar | Papad Fried | |
| 39 | | | Direct oral sugar intake | Pickle | |
| 40 | | | Jam | Boiled egg | |
| 41 | | | Jaggery | Bhurji | |
| 42 | | | Gur | Omlette | |

**Micro-nutrients, vitamins and minerals estimation using pure study software**

*Example:*

For example, if a participant has consumed two paranthas and a glass of milk in a day, their average portion size for parantha is two, and their frequency is one. For milk, portion size is one glass and frequency is one. When this data is entered into this software, the software estimates the nutrients based on the raw ingredients that are usually used for the preparation of the parantha. The software uses the information provided for raw ingredients for parantha as wheat flour, potatoes, oil, and spices. Based on the Indian dietary data provided by the National Institute of Nutrition, the software uses the weight of wheat flour, potato and oil in the two paranthas and a glass of milk. Further, as NIN provides data on the nutrients present per 100 g of food ingredients, the software uses the nutrients data given by NIN as per the quantity of ingredients used in preparation of the parantha. In two paranthas, it will estimate the result for proteins, fats and salt present in the ingredients of two paranthas and a glass of milk.

<table>
<tr><td colspan="3" rowspan="2">Breakfast</td><td colspan="2" rowspan="2">Estimated raw weight</td><td colspan="4">Calculated nutrient intake per 100 gram</td></tr>
<tr><td>Protein (g)</td><td>Carbohydrates (g)</td><td>Fat (g)</td><td>Energy (Kcal</td></tr>
<tr><td rowspan="2">Food Item</td><td rowspan="2">Stuffed parantha</td><td rowspan="2">Milk (I glass)</td><td rowspan="5">2 stuffed parantha</td><td>Wheat Flour =50g</td><td>5<br>2.5</td><td>33<br>16.5</td><td>0.7<br>0.35</td><td>158<br>79</td></tr>
<tr><td rowspan="2">Potato = 40g</td><td rowspan="2">0.6<br>0.24</td><td rowspan="2">9<br>3.6</td><td rowspan="2">0.4<br>0.16</td><td rowspan="2">42<br>16.8</td></tr>
<tr><td rowspan="2">Avg. Portion<br>(quantity in which a food item is eaten at a time)</td><td rowspan="2">2</td><td rowspan="2">1</td></tr>
<tr><td rowspan="2">Oil=10ml</td><td rowspan="2"></td><td rowspan="2">53<br>5.3</td><td rowspan="2">9<br>0.9</td><td rowspan="2">293<br>29.3</td></tr>
<tr><td rowspan="3">No. of times<br>(no. of times a particular food item is eaten in a day)</td><td rowspan="3">1</td><td rowspan="3">1</td></tr>
<tr><td colspan="2">1 glass milk=150ml</td><td>20<br>30</td><td>12<br>18</td><td>34<br>51</td><td>434<br>651</td></tr>
<tr><td colspan="2">Total</td><td>25.6<br>32.74</td><td>107<br>43.4</td><td>44.1<br>52.41</td><td>927<br>776</td></tr>
</table>

**Salt estimation**

***Salt***

Sodium is estimated using methodology mentioned above. Sodium estimated through the PURE study software was in mg/d. Sodium was convereted into salt g/d by using methodology adopted by previous studies[97]

Where,

Salt g/d= (Sodium mg/d × 2.54) ÷1000
