# Supplementary material for: Comparison of Measured 24-Hour Urinary Salt Excretion With Spot Urine and 24-Hour Dietary Recall Estimates Among Adolescents and Parents: Cross-Sectional Study
Source: JMIR Public Health Surveill. 2026 Jun 30;12:e85549. doi: 10.2196/85549 (PMC13317844; doi:10.2196/85549)
Supplement: Multimedia Appendix 9 [file publichealth-v12-e85549-s009.pdf]

APPENDIX S9: Estimation of Urinary Salt in g/d

24-hour salt g/d	Equations used
24-hour urine samples	***(((sodium in mmol/l)*23 *(urine volume in ml)/ 1000)*2.54)/1000)***
Spot urine samples	
Kawasaki (Male)	$2.54 \div 1000 \times 23 \times 16.3 \times$ (spot Na (mmol/l)/ [spot Cr (mg/dL) x 10] $\times$ [− 12.63 $\times$ age (years) + 15.12 $\times$ weight (kg) +7.39 $\times$ height (cm) – 79.9])$^{0.5}$
Kawasaki (Female)	$2.54 \div 1000 \times 23 \times 16.3 \times$ (spot Na (mmol/l)/[spot Cr (mg/dL) x 10] $\times$ [− 4.72 $\times$ age (years) + 8.58 $\times$ weight (kg) + 5.09 $\times$ height (cm) − 74.5])$^{0.5}$
Tanaka	$2.54 \div 1000 \times 23 \times 21.98 \times$ (spot Na (mmol/l)/[spot Cr (mg/dL) $\times$ 10] $\times$[− 2.04 $\times$ age (years) + 14.89 $\times$ weight (kg) + 16.14 $\times$ height (cm) − 2244.45])$^{0.392}$
INTERSALT with Potassium (Male)	$2.54 \div 1000 \times 23 \times$ (25.46 + [0.46 $\times$ spot Na (mmol/L)] – [2.75 $\times$ spot Cr (mmol/L)] − [0.13 $\times$ spot K (mmol/L)] + [4.10 $\times$ BMI (kg/m^2)] + [0.26 $\times$ age (years)])
INTERSALT with Potassium (Female)	.54 $\div$ 1000 $\times$ 23 $\times$ (25.46 + [0.46 $\times$ spot Na (mmol/L)] – [2.75 $\times$ spot Cr (mmol/L)] − [0.13 $\times$ spot K (mmol/L)] + [4.10 $\times$ BMI (kg/m^2)] + [0.26 $\times$ age (years)])
INTERSALT without Potassium (Male)	$2.54 \div 1000 \times 23 \times$ (23.51 + [0.45 $\times$ spot Na (mmol/L)] – [3.09 $\times$ spot Cr (mmol/L)] + [4.16 $\times$ BMI (kg/m^2)] + [0.22 $\times$ age (years)])
INTERSALT without Potassium (Female)	$2.54 \div 1000 \times 23 \times$ (3.74 + [0.33 $\times$ spot Na (mmol/L)] – [2.44 $\times$ spot Cr (mmol/L)] + [2.42 $\times$ BMI (kg/m^2)] + [2.34 $\times$ age (years) − [0.03 $\times$ age^2(years)])

Note: All lab values were initially in mmol/L \| 24-hour urine volume was noted in ml
